# Caspase-3 Mediates the Pathogenic Effect of *Yersinia pestis* YopM in Liver of C57BL/6 Mice and Contributes to YopM's Function in Spleen

**DOI:** 10.1371/journal.pone.0110956

**Published:** 2014-11-05

**Authors:** Zhan Ye, Amanda A. Gorman, Annette M. Uittenbogaard, Tanya Myers-Morales, Alan M. Kaplan, Donald A. Cohen, Susan C. Straley

**Affiliations:** Department of Microbiology, Immunology, and Molecular Genetics, University of Kentucky, Lexington, KY, United States of America; University of Helsinki, Finland

## Abstract

The virulence protein YopM of the plague bacterium *Yersinia pestis* has different dominant effects in liver and spleen. Previous studies focused on spleen, where YopM inhibits accumulation of inflammatory dendritic cells. In the present study we focused on liver, where PMN function may be directly undermined by YopM without changes in inflammatory cell numbers in the initial days of infection, and foci of inflammation are easily identified. Mice were infected with parent and Δ*yopM-1 Y. pestis* KIM5, and effects of YopM were assessed by immunohistochemistry and determinations of bacterial viable numbers in organs. The bacteria were found associated with myeloid cells in foci of inflammation and in liver sinusoids. A new in-vivo phenotype of YopM was revealed: death of inflammatory cells, evidenced by TUNEL staining beginning at d 1 of infection. Based on distributions of Ly6G^+^, F4/80^+^, and iNOS^+^ cells within foci, the cells that were killed could have included both PMNs and macrophages. By 2 d post-infection, YopM had no effect on distribution of these cells, but by 3 d cellular decomposition had outstripped acute inflammation in foci due to parent *Y. pestis*, while foci due to the Δ*yopM-1* strain still contained many inflammatory cells. The destruction depended on the presence of both PMNs in the mice and YopM in the bacteria. In mice that lacked the apoptosis mediator caspase-3 the infection dynamics were novel: the parent *Y. pestis* was limited in growth comparably to the Δ*yopM-1* strain in liver, and in spleen a partial growth limitation for parent *Y. pestis* was seen. This result identified caspase-3 as a co-factor or effector in YopM's action and supports the hypothesis that in liver YopM's main pathogenic effect is mediated by caspase-3 to cause apoptosis of PMNs.

## Introduction

The Gram-negative bacterium *Yersinia pestis* is a vector-borne pathogen that causes plague in humans. This disease manifests in three forms, bubonic due to injection of the bacteria into the dermis by the flea vector, pneumonic due inhalation of an infectious aerosol, and septicemic due to systemic dissemination within the body [1]. YopM is among a set of virulence proteins termed Yops that function within the mammalian host. Their genes are thermally upregulated: in the flea vector at ambient temperature they are expressed at low basal levels, whereas they are strongly expressed once within mammalian tissues. Additional thermally upregulated virulence properties that function alongside and in conjunction with the Yops are adhesins, anti-phagocytic fibrils, and an under-acylated lipooligosaccharide that evades pro-inflammatory signaling through Toll-like receptor 4 (TLR4) [2]–[4]. The Yops are delivered to the cytosol of mammalian cells by a type 3 secretion system (T3SS). There they inhibit signaling pathways involved in phagocytosis and development of inflammation [5]. Overall, the Yops function early in infection to counteract innate defenses and thereby promote bacterial replication to the overwhelming numbers needed for transmission to a flea vector in a blood meal.

YopM has a leucine-rich repeat structure without obvious enzymatic domains and is believed to function as a scaffold that binds host proteins [6], [7]. Two serine/threonine kinases, ribosomal protein S6 kinase one (RSK1 or p90RSK) and protein kinase C-like (PRK or PKN) are bound to YopM in large complexes and, despite being activated in the complex, fail to activate their usual downstream targets [7]–[9]. YopM also inhibits the cysteine protease caspase-1. This prevents inflammasome maturation and limits release of the pro-inflammatory cytokines IL-1 and IL-18 [10]. YopM's effect on either RSK1 or caspase-1 promotes virulence of the bacteria [9], [10]; however it is not known if or how these effects are linked biochemically. Interactions of YopM with serum proteins have been documented; but these have not been found to have significant effects on virulence, likely because most of YopM is delivered into cells without release into extracellular fluid [11]–[14]. Pure YopM can promote its own entry into cells [15]. Once inside either spontaneously or via the T3SS, YopM traffics to the nucleus in a process that is promoted by vesicular trafficking [14], [15]. Accordingly, YopM's effects could involve multiple host molecules in both cytoplasm and nucleus.

In a mouse model of systemic plague, intravenously (IV) injected *Y. pestis* KIM5 seeds liver and spleen within 30 minutes [16] and replicates. If the strain lacks YopM, a host response curbs net growth beginning ∼ d 2 post-infection (p.i.). In infected spleen, that time point marks the beginning of a progressive qualitative shift in populations of inflammatory cells [17], [18]. Mice infected with the parent YopM^+^ *Y. pestis* KIM5 showed loss of inflammatory MOs and iDCs as well as natural killer (NK) cells from spleen in contrast to ones infected with Δ*yopM-1 Y. pestis*, where these cells accumulated in spleen [17]–[19]. The critical cells for controlling Δ*yopM-1 Y. pestis* in spleen proved to be the inflammatory MOs and DCs, not NK cells or PMNs, which were dispensable for limiting growth of the bacteria in spleen [18], [19]. In contrast to spleen, livers of *Y. pestis*-infected mice did not show YopM-associated differences in recruitment of MOs, iDCs, or NK cells. Recruitment of PMNs also was not affected by YopM; however, PMNs were critical for controlling growth of Δ*yopM-1 Y. pestis* in liver [18], [19]. It was hypothesized that in liver, YopM inhibits PMN antibacterial function. Further, when parent *Y. pestis* was co-infected with Δ*yopM-1 Y. pestis*, the parent strain protected the mutant from growth limitation in spleen but not in liver [18]. These results were consistent with the interpretation that in spleen YopM prevents accumulation of a mobile component that is anti-bacterial (iDCs), whereas in liver, the bacteria act directly, one-on-one, against antibacterial host cells [18], [19]. Accordingly, YopM has different critical effects in the two organs.

Previous studies on YopM function have emphasized spleen over liver; yet liver has the advantage of a highly regular architecture within which inflammatory cells are easily distinguished; and the populations of inflammatory cells do not differ between infections by parent and Δ*yopM-1 Y. pestis*. We report here studies that revealed a pathogenic effect of YopM unique to liver and an element of a pathway in which YopM functions.

## Materials and Methods

### Ethics statement

All animal experiments in this study were reviewed and approved by the University of Kentucky Institutional Animal Care and Use Committee. The protocol under which the work was conducted was Number 2007–0077, “YopM and Protective Innate Defenses against Plague”. Mice were anesthetized during infection procedures, infected mice were checked twice daily for signs of illness, and they were humanely killed by CO_2_ inhalation followed by cervical luxation. They were not allowed to die of plague.

### Bacterial strains and *in vitro* cultivation

This study employed *Y. pestis* KIM5 (molecular grouping 2.MED), an isolate from Iran, and the Δ*yopM-1* derivative KIM5-3002. They lack the chromosomal *pgm* locus and are conditionally virulent: attenuated from peripheral routes of infection but virulent by the IV route of infection, which bypasses the requirement for a siderophore-based iron-acquisition mechanism encoded in this locus [20]. Two growth protocols were used for *Y. pestis* KIM5-derived strains. The bacteria were grown at 28°C for at least 6 generations at exponential phase in Heart Infusion Broth (HIB, Difco Laboratories) and harvested in exponential phase at A620 = 0.8 to 1.2 or, after 6 generations in exponential phase at 28°C they were transferred to 37°C at A620 of ca. 0.3 and incubated for 3 h (28/37°C). For infection of mice, cells were centrifuged at 23,000×g and 4°C for 5 min and then washed and diluted in phosphate-buffered saline (PBS). Samples from the dilutions were plated on tryptose blood agar (Difco Laboratories) and incubated at 30°C for 2 d to determine the actual dose.

### Infection of mice and measurement of bacterial viable numbers in tissues

All mice used were female and 6–12 weeks of age. The majority of experiments used C57BL/6N.HSD mice (B6; Harlan Sprague Dawley, Inc). Some experiments used transgenic mouse strains on a C57BL/6 background (The Jackson Laboratory): mice lacking inducible nitric oxide synthase (iNOS) (B6.129P2-*Nos2^tm1Lau^*/J) and mice lacking caspase-3 (Casp3^−/−^) (B6.129S1-*Casp3^tm1Flv^*/J). Mice were anesthetized with an isoflurane-oxygen mixture by a rodent anesthesia machine. The specified number of CFU of *Y. pestis* in 50 µL was injected in each mouse IV via the retro-orbital plexus. At designated times after infection, groups of mice were humanely killed by CO_2_ inhalation followed by cervical luxation, and their spleens and livers were transferred to a sterile bag containing 5 mL of PBS and homogenized in a Stomacher 80 lab blender (Tekmar Co.). When portions of livers were to be used for histology and the rest for determination of viable bacterial numbers, the livers were handled singly. Each liver was weighed, the portion for histology was removed, and the remainder was weighed again and homogenized. The liver and spleen homogenates were diluted in pH 11 water followed by further dilutions in PBS. Samples were plated to measure viable bacterial numbers (CFU) in the organs, corrected as necessary for removed portions of livers.

Because we used bacteria pre-grown at 28/37°C in addition to 28°C in some experiments we determined the extent to which thermal pre-induction affected virulence. The LD_50_ values were measured for 28°C- and 28/37°C-grown Δ*yopM-1 Y. pestis* KIM5-3002 in B6 mice. An initial test with 10-fold dilutions of bacteria established the dose range to be used. Then groups of 8 mice were given serially 3-fold diluted bacterial doses. The mice were observed twice daily for mortality for 14 d. Mice that had become prostrate and deemed, from experience, not likely to survive to the next observation were humanely killed, and their death was recorded as of the following observation. The LD_50_ was determined by probit analysis of the survival data. The LD_50_ of the 28/37°C-grown Δ*yopM-1* mutant was 5±2×10^4^, ca. 4-fold lower than that for the same strain grown at 28°C (2±1×10^5^). This result confirmed that there was a measurable but small difference in lethality due to the incubation at 37°C.

### PMN ablation

PMNs were ablated with the PMN-specific anti-Ly6G mAb (mAb 1A8; Bio × Cell); control mice received rat IgG (Sigma-Aldrich Co.). The antibodies were injected intraperitoneally as 200 µg/mouse in 100 µL PBS on d –1 and +1 of *Y. pestis* infection. The extent of ablation in these and in mock-treated mice on d 1 to d 3 p.i. was shown to be 88% to 99% by flow cytometry with detection via fluorochrome-tagged anti-Ly6G and anti-CD11b antibodies (see below).

### Flow cytometry

Leukocytes were analyzed in a FACSCalibur flow cytometer (Becton Dickinson FACS Systems) as described previously [18]. Briefly, splenic leukocytes were centrifuged and resuspended in 2 mL RBC lysis buffer (150 mM NH_4_Cl, pH 7.2). After 2 min, the lysis was terminated by addition of 8 mL of PBS. The cell suspensions were filtered through a 40 µm-pore-size cell strainer (Becton, Dickinson and Company), centrifuged, and resuspended in PBS containing 1% (wt/vol) BSA and 0.1% (wt/vol) sodium azide (PBS-BSA-azide). Minced livers in 10 mL RPMI 1640 (Life Technologies Corp.) with 10% fetal bovine serum (FBS; ATCC) were processed with a Stomacher 80 lab blender. Samples were removed to plate for CFU, and the remaining homogenates were incubated with DNase I (160 U/mL, Sigma-Aldrich) and collagenase (400 U/mL, Life Technologies) for 30 min at 37°C with shaking at 125 rpm. After filtration through a 40 µm-pore-size cell strainer, cell suspensions were washed once with ice-cold PBS-BSA-Azide and resuspended with 5 mL 33.8% Percoll (Amersham bioscience, GE Healthcare) followed by centrifugation. The cells in the bottom layer were collected, washed with PBS, treated with RBC lysis buffer, and resuspended in PBS-BSA-azide.

The following antibodies were purchased from BD Pharmingen, Inc.: allophycocyanin (APC)-conjugated anti-CD11c, Fluorescein isothiocyanate (FITC)-conjugated anti-CD11b, Phycoerythrin (PE)-conjugated anti-Ly6G, and Fc Block (Rat Anti-Mouse CD16/CD32). To gate out apoptotic or dead cells, ethidium monoazide (EMA, Sigma-Aldrich) was used for viability staining.

Prior to staining, the cells were counted, and samples were adjusted to 5×10^6^ cells per mL. Then they were stained as described previously [18]. Briefly, after treatment with Fc Block for 15 min on ice, the cells were incubated with appropriate combinations of antibodies and EMA on ice for 30 min, with the last 10 min of incubation being in the light. After being washed in PBS, they were resuspended in ice-cold PBS containing 4% (wt/vol) paraformaldehyde (pH 7.4) for at least 30 min before flow cytometric analysis.

Flow cytometric processing was performed by the Flow Cytometry Core Facility at the University of Kentucky. The data were analyzed with WinMDI (Version 2.7; Joseph Trotter, Salk Institute for Biological Studies, La Jolla, CA [available at http://facs.Scripps.edu/software.html]).

### Immunohistochemistry for cell-surface markers

Livers and spleens were nicked several times with a scalpel blade, fixed overnight in 10% (vol/vol) buffered formalin (Fisher Scientific Inc.) or Histochoice MB (Electron Microscopy Sciences), and transferred to 70% (vol/vol) ethanol. They were dehydrated and paraffin-embedded by Cynthia Long in the University of Kentucky Imaging Core Facility. 5-µm-thick sections were cut, deparaffinized with xylene, rehydrated through a graded ethanol series, and stained with hematoxylin and eosin (H&E). The general immunohistochemistry (IHC) procedure was as follows. Deparaffinized sections were rinsed in tap water 1 min and submerged 20 min in hot citrate buffer (5 mM Na citrate.2H_2_O+1% [vol/vol] Tween 20 pH 6.0) in a coplin jar within a vegetable steamer (antigen retrieval step). The coplin jar was removed to room temperature and allowed to stand, uncapped, 30 min. The citrate buffer in the jar was replaced, and the slides were rinsed with warm water for several minutes (more than 2 complete changes) and then treated with 3% (vol/vol) H_2_O_2_ 5 min to saturate background peroxidases. Slides were rinsed with PBS, incubated in PBS 5 min, and blocked 1 h at room temperature (PBS + FcBlock +10% [vol/vol] normal serum [NS] +1% [wt/vol] histochemical grade BSA [Vector Laboratories]). Sections were then covered with primary antibody (1^o^ Ab; in PBS +1% [wt/vol] histochemical grade BSA) and incubated overnight at 4°C in a humidor. If the 2^o^ Ab was from rat, it was first adsorbed for 30 min at 4°C with 1% (wt/vol) acetone powder prepared from mouse livers and spleens and then clarified by centrifugation. The slides were given two 5-min washes with water and stained with biotinylated secondary antibody (2^o^ Ab) 30 min at room temperature. They were stained by using Vectastain ABC (avidin/biotinylated enzyme complex; Vector), developed with 3, 3′-diaminobenzidine (DAB; Sigma-Aldrich), counterstained 1 min with hematoxylin QS (Vector), rinsed 1 min in water (until water ran clear), cleared through ethanol and xylenes, and mounted. Protocol details were optimized for each antibody and organ. For F4/80, blocking NS was rabbit (Invitrogen Corp./Life Technologies), 1^o^ Ab was rat monoclonal BM8 (eBioscience) diluted 1∶500 (liver) or 1∶800 (spleen), and 2^o^ Ab was mouse-adsorbed biotinylated anti-rat IgG (Vector Laboratories) diluted 1∶100. For iNOS, blocking NS was goat (Invitrogen Corp./Life Technologies), 1^o^ Ab was rabbit polyclonal anti-iNOS (Abcam; cat. No. ab15323) diluted 1∶150 (liver) or 1∶200 (spleen), and 2^o^ Ab was biotinylated anti-rabbit IgG (Vector Laboratories) diluted 1∶250. For Ly6G, blocking NS was rabbit, 1^o^ Ab was rat monoclonal 1A8 (BD Pharmingen) diluted 1∶1000 (liver) or 1∶1500 (spleen), and 2^o^ Ab was mouse-adsorbed biotinylated anti-rat IgG diluted 1∶250. For “capsular” antigen F1 on *Y. pestis*, blocking NS was goat, 1^o^ Ab was mouse monoclonal YPF19 (Abcam cat. No. ab8275) diluted 1∶100, and 2^o^ Ab was biotinylated anti-mouse IgG (Vector Laboratories) diluted 1∶250. We also used as 1^o^ Ab a rabbit anti-37°C-grown *Y. pestis* KIM6-whole-cell antibody diluted 1∶1000 (liver) or 1∶250 (spleen) with biotinylated anti-rabbit IgG 2^o^ Ab diluted 1∶250 and obtained similar results. Histochoice fixation resulted in better staining for F4/80 and iNOS than fixation by formalin.

TUNEL assays were carried out on formalin-fixed paraffin-embedded sections as follows. Deparaffinized sections were rinsed in tap water 1 min and submerged 10 min in hot citrate buffer (5 mM Na citrate.2H_2_O +1% [vol/vol] Tween 20 pH 6.0) in a coplin jar within a vegetable steamer (antigen retrieval step). The coplin jar was removed to room temperature, 25 ml of room-temperature PBS was added, and the slides were immediately transferred to fresh PBS for 5 min followed by a second 5-min soak in PBS. Slides were then blocked 1 h at room temperature (PBS +20% [vol/vol] heat-inactivated FBS +1% [wt/vol] BSA). They were washed by two 2-min changes of PBS, liquid drops were removed withabsorbent paper, and then sections were covered with rat anti-BrdU (1^o^ Ab; Abcam Ab6236) diluted 1∶40 in the Strep Dilution Buffer from the Trevigen TACS XL *in situ* apoptosis detection kit and incubated overnight at 4°C in a humidor. The slides were given two 5-min changes of PBS, liquid drops were blotted, and then sections were covered with mouse-adsorbed biotinylated rabbit anti-rat IgG (2^o^ Ab) diluted 1∶250 in Strep Dilution Buffer or PBS +1% BSA and incubated 2 h at room temperature in a humidor. Slides were given three 5-min changes of PBS. Subsequent steps were performed in the dark. Sections were covered with Oregon green-conjugated neutravidin (Invitrogen Molecular Probes) diluted 1∶250 in Strep Dilution Buffer and incubated 1 h at room temperature in a humidor. The slides were given three 5-min soaks in PBS, blotted, mounted with Vectashield (Vector) containing 0.143 mM 4′, 6-diamidino-2-phenylindole (DAPI), and sealed with nail polish.

Sections were examined with a Nikon Eclipse E800 microscope and photographed with a Photometrics CoolSNAP cf camera. Sections were scanned and fields were photographed systematically. Size markers were inserted into images by the Nikon NIS Elements v 3.22 software. These aided measurements of the widest aspect of inflammatory foci for purposes of classification into arbitrary categories of small, medium, and large (<50 µm, 50–100 µm, and>100 µm, respectively). In tissue sections stained by the TUNEL reaction, foci were first identified by the presence of densely clustered nuclei stained with DAPI, and then TUNEL staining was recorded. TUNEL-stained foci were analyzed for the percent of area occupied by staining over a threshold value that excluded nonspecific background staining by using MetaMorph v. 5.0 r7 software (Universal Imaging Corp.). Figures were composed with Adobe Photoshop CS5.

### Statistical analysis

Infection experiments were conducted at least twice with at least 3 replicate mice per group. Supportive data are given in Table S1 and Figure S1. The numbers of mice used for each datum point are given in the figure legends. Significance of differences among groups was assessed by the Student's 2-tailed unpaired *t* test. Values for P<0.05 were considered significant. The LD_50_ and 95% confidence intervals were determined from the probit analysis of the mortality data (Biostat software, Analystsoft.com). Categorical distributions of cells within lesions were compared for difference by using a 2-tailed Fisher's exact test, which determines whether the odds ratio is significantly different from 1. P Values <0.05 were considered significant.

## Results and Discussion

### Cells directly associated with *Y. pestis* in liver

**Figure 1:**
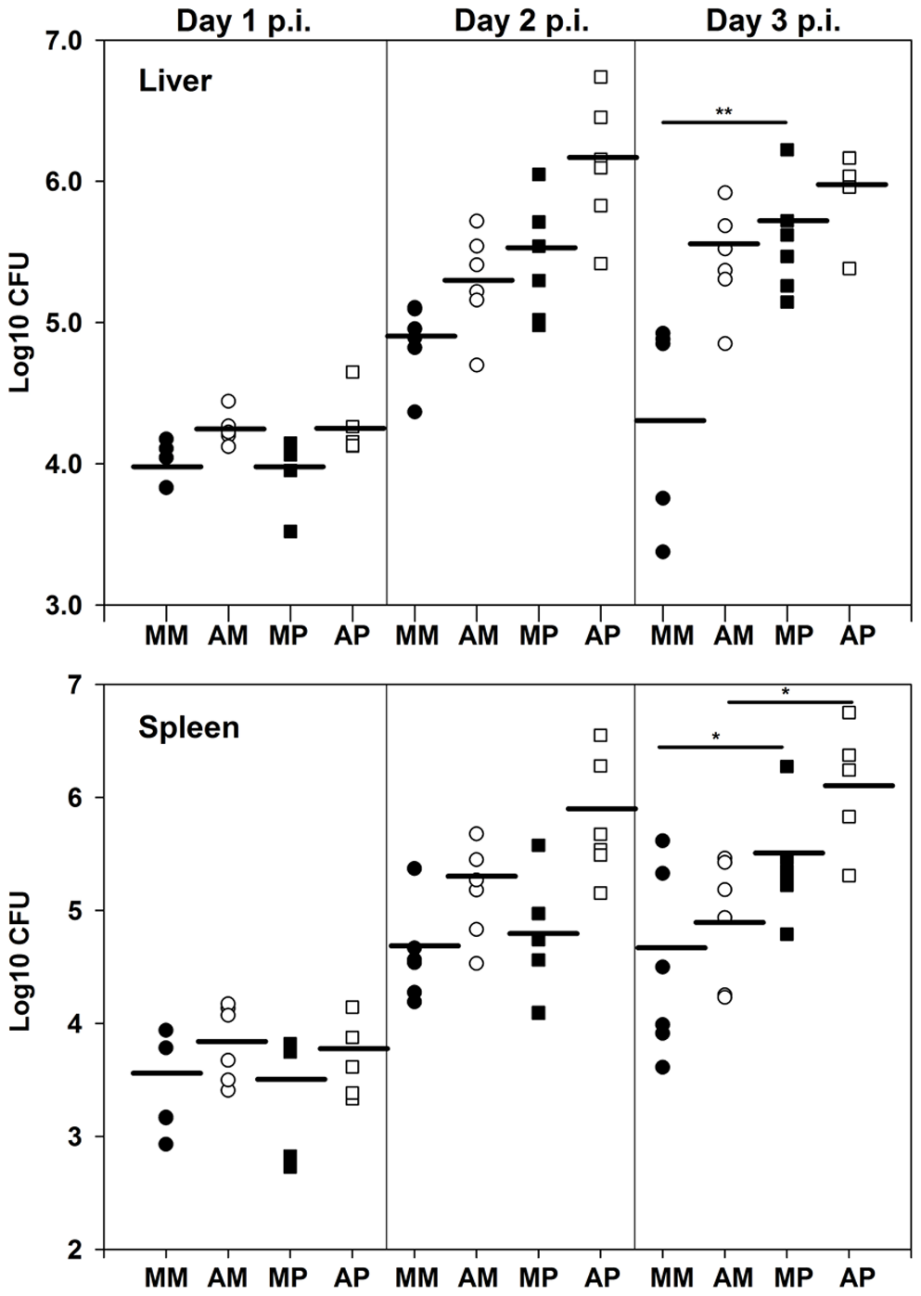
PMNs were critical to control growth of 28/37°C-pregrown Δ*yopM-1 Y. pestis* in liver but not spleen. B6 mice were ablated for PMNs (open symbols) by treatment with anti-Ly6G antibody on days -1 and +1 of infection; mock-treated mice (closed symbols) received nonspecific rat IgG. The mice were infected with 400 28/37°C-grown parent (squares) or Δ*yopM-1* (circles) *Y. pestis*, and viable numbers (CFU) were determined on d 1, 2, and 3 p.i. Group labels: MM, mock-treated mice infected with Δ*yopM-1 Y. pestis*; AM, Ablated mice infected with Δ*yopM-1 Y. pestis*; MP, mock-treated mice infected with the parent strain; AP, ablated mice infected with the parent strain. The data were obtained in two experiments, each with 2 to 3 mice per group, for 4 to 6 mice total for each category. Each symbol gives the bacterial burden of one mouse; the horizontal lines mark the geometric means of the pooled data for each category. Statistically significant differences by Student's *t* test occurred on d 3 p.i. as indicated: *, P<0.05; **, P<0.01.

**Figure 2:**
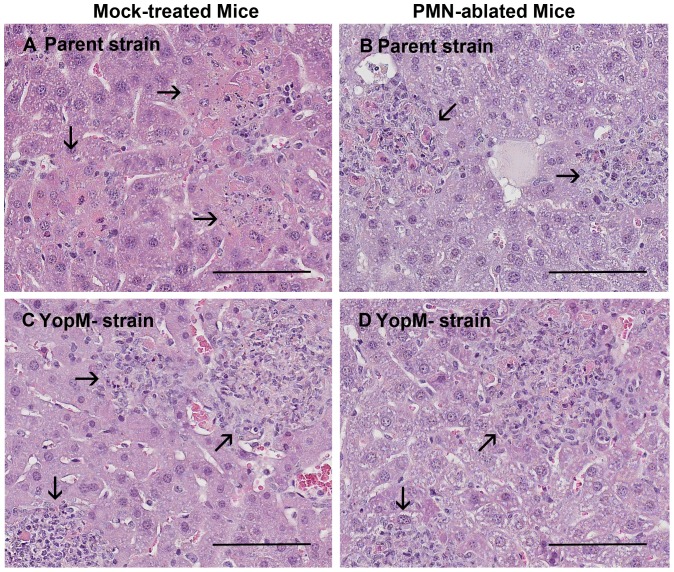
Destruction of inflammatory cells correlated with the presence of both YopM and PMNs. Portions of livers from all mice in one of the experiments of Figure 1 were fixed in formalin, sectioned, and stained with H&E. Lesions are illustrated for d 3 p.i. in mice with bacterial burdens similar to the means of the pooled data of Figure 1. Panel A, mock-treated mouse infected with parent *Y. pestis* (2×10^6^ CFU in liver); panel B, PMN-ablated mouse infected with parent *Y. pestis* (1×10^6^ CFU in liver); panel C, mock-treated mouse infected with Δ*yopM-1 Y. pestis* (7×10^4^ CFU in liver); panel D, PMN-ablated mouse infected with Δ*yopM-1 Y. pestis* (5×10^5^ CFU in liver). Arrows point to inflammatory lesions. The bars represent 100 µm.

We had shown that PMNs are critical for control over growth of 28°C-grown Δ*yopM-1 Y. pestis* in liver [19]. In this study we verified that this also holds when the bacteria were grown at 28/37°C (Figure 1). In one of the experiments of Fig. 1, we evaluated the histology in each of the 3 mice per treatment group on each of the three days. Figure 2 illustrates typical lesions for d 3 p.i. in mice with bacterial burdens near to the means of the pooled data of Figure 1. In mock-treated mice, parent *Y. pestis* formed compact coagulatively necrotic lesions with only bits of inflammatory cells remaining (Fig. 1A), while foci due to the Δ*yopM-1* strain in mock-treated mice were larger and retained many intact cells (panel C). In mice ablated for PMNs (panels B and D), the lesions due to both strains were similar to ones due to Δ*yopM-1 Y. pestis* in mock-treated mice. These findings are consistent with the critical involvement of PMNs in cellular destruction as previously found in pneumonic plague, where PMNs were shown to be responsible for the architectural damage and major symptoms of disease [21]. Further, it was striking that the extensive destruction of inflammatory cells was seen only when the bacteria expressed YopM and the mice had PMNs (Fig. 2 panel A), even though similarly high bacterial numbers were present in livers of the PMN-ablated mice infected with parent *Y. pestis* (panel B).

**Figure 3:**
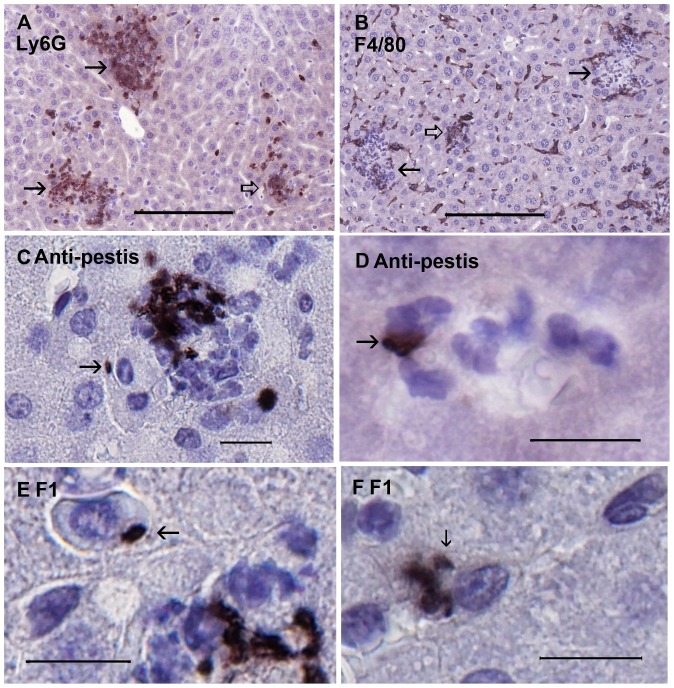
Early in infection many *Y. pestis* KIM5 bacteria were associated with inflammatory cells including PMNs. Mice were infected IV for 17 h with 10^7^ 28/37°C-grown *Y. pestis* KIM5. Sections of formalin-fixed paraffin-embedded livers were stained with cell- or *Y. pestis*-specific antibody, developed with DAB (black or brown), and counterstained with hematoxylin. Antibodies used were: Panel A, anti-Ly6G (for PMNs); Panel B, anti-F4/80 (for KCs and MOs); Panels C and D, anti-*Y. pestis* antibodies; Panels E and F, the YPF19 monoclonal antibody against the F1 fibril. Solid arrows in panels A and B indicate foci of inflammation; open arrows indicate either small foci or “polar cap” (tangential) sections of foci. The arrow in panel C points to a bacterium on a mononuclear cell adjacent to a focus where numerous bacteria were contained in the plane of the section. The arrow in panel D indicates several bacteria associated with PMNs in a hepatic sinus. Arrows in panels E and F point to bacteria associated with mononuclear cells. The bars in panels A and B represent 100 µm; in panels C-F, the bars represent 10 µm.

Our previous studies had indicated that in liver YopM undermines PMN antibacterial function without a diffusible mediator [19]. This could occur through direct binding and delivery of YopM to PMNs or through delivery of YopM to a cell such as a KC that PMNs must directly interact with to have their antibacterial effect. To establish the context in which YopM functions early, we used IHC to identify inflammatory cells and locate *Y. pestis* in livers of B6 mice infected for 17 h with thermally pre-induced *Y. pestis* KIM5 at a dose high enough (10^6^ or 10^7^) that bacteria would be found in thin sections. Figure 3A shows that these early inflammatory foci were populated by PMNs, which tended to be evenly distributed as illustrated (based on nuclear morphology in H&E-stained sections from 4 experiments and Ly6G staining in the experiment of Figure 3: see Table S1). F4/80^+^ cells (Kupffer cells [KCs] and MOs) were scattered in sinusoids and clustered around foci (Fig. 3B). If the focus was small, they tended to be present over the entire focus (∼70% of such foci), but in larger foci they were restricted to the periphery (∼84% of such foci) (quantification is given in Table 1 and Table S1). This observation indicates that as foci enlarge, KC/MOs are lost from the centers.

**Table 1:** Distribution of F4/80^+^ cells in inflammatory foci after infection with thermally preinduced *Y. pestis.*

F4/80^+^ Cell Distribution within Foci^a^								
		Foci	Small Foci^b^		Medium Foci^b^		Large Foci^b^	
Dose; Time; (No. Exps)	*Y. pestis*	Total No.	Central^c^	Edge^c^	Central	Edge	Central	Edge
10^6^ 17 h (2^d^)	Parent	116	69% (51)	31% (23)	17% (6)	83% (30)	17% (1)	83% (5)
	Δ*yopM-1*	122	77% (53)	23% (16)	18% (9)	82% (41)	0% (0)	100% (3)

a Within each focus size, the percent having each distribution of stained cells is given, and the actual number of foci in each category is in parentheses.

b Foci were measured in their largest aspect and classified as small (<50 µm), medium (50 to 100 µm), and large (>100 µm).

c The presence of stained cells within the central part of the focus identified that lesion as having a Central distribution. Foci with a central distribution of stained cells tended to have F4/80^+^ cells all over the focus (not just at the center). If the cells were seen only in a ring around the perimeter extending inward not more than 25% of the diameter of the focus, the distribution was designated as Edge.

d Data are from two mice per infecting *Y. pestis* strain, one from each independent experiment. The distributions between small vs. medium + large foci for 17-h infections by the two *Y. pestis* strains (the two experiments with dose  = 10^6^) did not differ significantly by Fisher's exact test. For both parent and Δ*yopM-1 Y. pestis* in both 10^6^ and 10^7^ doses, the distributions of F4/80^+^ cells between small vs. medium + large foci were statistically different by Fisher's exact test (p = 0.000 for 2-tailed determination that the odds ratio is significantly different from one, where the odds ratio  =  No. Small Central/No. Small Edge ÷ No. Medium+Large Central/No. Medium+Large Edge).

**Figure 4:**
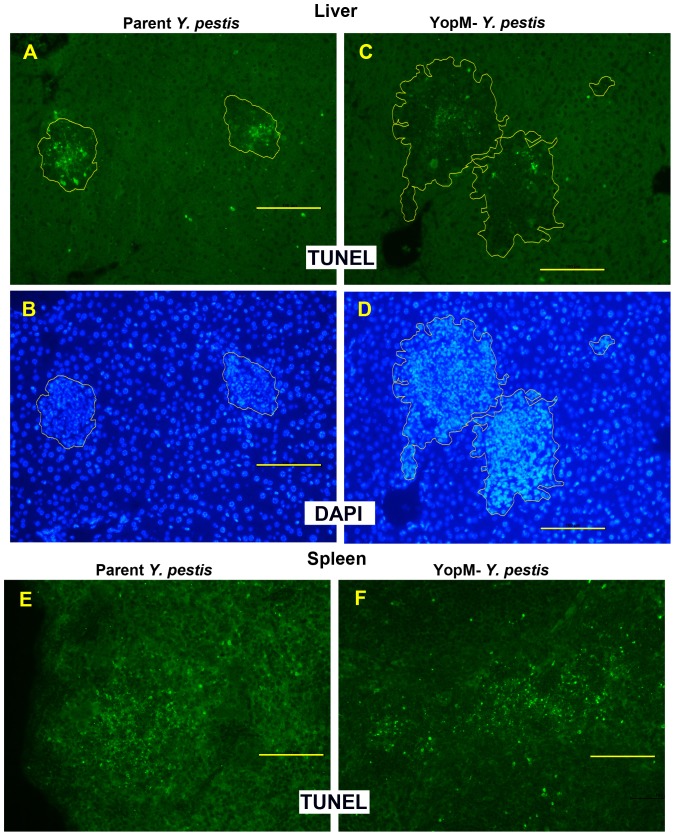
YopM caused death of inflammatory cells in liver. Mice were infected 2 d with 3×10^4^ thermally pre-induced parent *Y. pestis* KIM5 or the Δ*yopM-1* strain. Liver and spleen sections were stained by the TUNEL reaction to reveal cells undergoing DNA fragmentation. Panels A, C, E, and F show TUNEL^+^ staining by bright green fluorescence. Panels B and D show the same sections as panels A and C stained for DNA by DAPI (blue fluorescence) to reveal the distribution of cells by their nuclei. Foci were identified based on such nuclear accumulations. The boundaries of the foci were outlined on the DAPI images using tools within the morphometrics software and transferred to the corresponding positions in the TUNEL images. The areas within the outlines were used by the morphometrics program for quantification of TUNEL staining. For this illustration, the outlines were traced within the Photoshop program to make them 5 pixels thick. The bars represent 100 µm.

**Figure 5:**
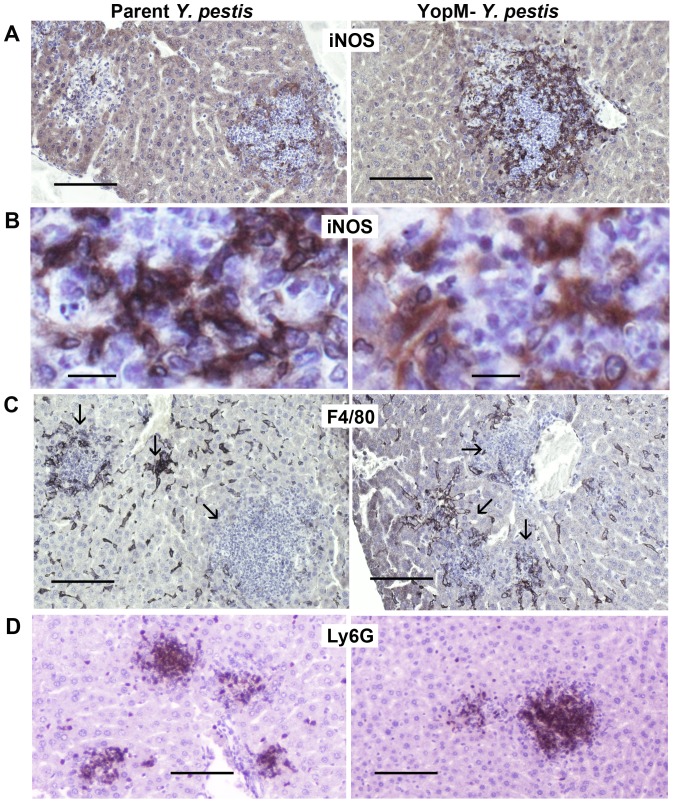
YopM did not affect the distributions of inflammatory cells. Liver sections from the mice of Figure 3 were stained for inducible nitric oxide synthase (iNOS; panels A and B), the KC/MO marker F4/80 (panels C), or the PMN marker Ly6G (panels D). Antigen detection used DAB staining (black or brown) in sections from livers infected by parent *Y. pestis* (left panels) and the Δ*YopM-1* strain (right panels). The arrows in panels C point to inflammatory foci. The bars represent 100 µm (panels A, C, D) or 10 µm (panels B).

Within the foci, clusters of bacteria were associated with groups of inflammatory cells (Fig. 3C). In the sinusoids, bacteria were associated with PMNs (Fig. 3D) and mononuclear cells (KCs/MOs/DCs; Fig. 3C, E and F) and not with endothelial cells or hepatocytes. Similar observations were made at d 2 p.i. in mice infected with a lower dose as in Figures 4 and 5 (data not shown).

These observations show that *Y. pestis* interacts directly with myeloid cells in liver, including PMNs. Following IV infection, the bacteria would seed the liver via the hepatic artery, which mixes with the input of the hepatic portal vein and flows into the sinusoids to bathe the liver plate hepatocytes. The bacteria could bind to KCs that patrol the sinusoids and to PMNs that are rapidly attracted to the site. These are the first cells in liver that would be receiving YopM, consistent with findings for spleen, lung, and skin [21]–[23], showing that macrophages and PMNs are the earliest cells with which *Y. pestis* interacts.

### YopM promotes cell death

Our previous histopathologic observations on livers of mice 4 d after infection by a low dose (100) of 26°C-grown Δ*yopM-1 Y. pestis* found that in liver, inflammatory cells in foci remained morphologically intact longer than those due to the parent strain [17]. In the present study, mice were infected in two experiments that used doses ∼2-fold below the LD_50_ for Δ*yopM-1 Y. pestis* grown either at 28°C or 28/37°C. We stained infected tissues for presence of DNA strand breaks by terminal deoxynucleotidyl transferase dUTP nick end labeling (TUNEL). This assay will label cells in a late stage of apoptosis or pyroptosis. After infection with 10^5^ 28°C-grown parent or Δ*yopM-1 Y. pestis*, sections of livers and spleens from two mice for each strain were stained and quantified. On d 1 p.i., when lesions were small, virtually no TUNEL staining was observed in livers infected with the Δ*yopM-1* strain, whereas some staining was found for livers infected by the parent strain (data not shown). By d 2 p.i. when inflammatory foci were prominent, judging from the distributions of DAPI-stained nuclei, there was much more TUNEL staining in liver foci due to parent than Δ*yopM-1 Y. pestis*, (Fig. S1 panels A-D). The fraction of focus area occupied by TUNEL staining in foci due to parent *Y. pestis* was 8.0±4.8% (based on 21 foci), whereas foci due to the Δ*yopM-1* strain were 2.6±1.8% TUNEL^+^ (based on 32 foci), a difference that was statistically significant (2-tailed *t* test: P = 7.1×10^−7^). This assay was repeated on single mice infected 2 d with 3×10^4^ thermally induced parent or mutant *Y. pestis* with similar results (Fig. 4A-D): foci due to parent *Y. pestis* were 10.8±7.3% TUNEL^+^ (based on 44 foci), whereas those due to the Δ*yopM-1* strain were 3.9±3.6% TUNEL^+^ (based on 28 foci), again statistically significant (2-tailed *t* test: P = 1.6×10^−5^).

It was not possible to identify the TUNEL^+^ cells by their nuclear morphology, because TUNEL-staining nuclei were highly condensed or fragmented, and apoptotic PMN nuclei lose their multilobed structure [24]. However, the distribution of TUNEL^+^ nuclei was similar to the cellular distribution for PMNs and distinct from that for mononuclear cells. The TUNEL staining tended to be evenly distributed or centrally located (86%–93% of foci for the two strains in the two experiments). PMNs also tended to be evenly or centrally distributed in all sizes of foci. 90% of foci that contained TUNEL^+^ nuclei were medium to large, and staining only rarely was restricted to the edge (10% of TUNEL^+^ foci); whereas F4/80 cells were restricted to the edge of half to two-thirds of medium to large foci. These findings could be taken as support for the hypothesis that most of the TUNEL^+^ cells were PMNs that underwent cell death in response to YopM delivery in addition to some spontaneous apoptosis and phagocytosis-induced apoptosis [24]. However, the dead cells represented ones that had been present there earlier when the lesion was smaller, and F4/80^+^ cells were present uniformly in small foci along with PMNs. Hence, we cannot rule out a process in which F4/80^+^ cells initially were recruited but then killed as the lesion enlarged, resulting in peripherally-arranged F4/80^+^ cells in medium to large foci with centrally or uniformly distributed TUNEL staining. Accordingly in liver, infection with *Y. pestis* promotes YopM-associated death of inflammatory cells, probably both PMNs and KC/MOs, detectable as early as d 1 p.i. and prominent by d 2. TUNEL staining can result from oncosis and pyroptosis as well as apoptosis [24]; accordingly, further biochemical and cell biological assays will be required to identify which of these cell death mechanisms is stimulated by YopM in liver [25].

In spleen, similar amounts of TUNEL staining were seen for infections with parent and Δ*yopM-1 Y. pestis*, (illustrated in Fig. 4E and F). The foci were more clearly demarcated in mice infected with 10^5^ 28°C-pregrown yersiniae (Figure S1E and F), and quantification indicated nearly identical amounts of staining per focus, with the caveat that this was based on only 6 or 7 lesions for each infecting strain. Accordingly, YopM-associated cell death is a phenotype unique to liver. YopM may promote cell death in spleen as well as liver, although in spleen this activity apparently is redundant to a YopM-independent effect.

### iNOS^+^ cells invaded inflammatory foci by d 2 p.i

Because of the known importance of iMOs and iDCs for YopM's pathogenic effect in spleen, we evaluated the distribution of these cells in liver by staining for inducible nitric oxide synthase (iNOS), which these cells express strongly. Mice were infected with 10^4^ to 3×10^4^ thermally induced parent or Δ*yopM-1 Y. pestis*, and livers were obtained for IHC. In one of the experiments, one mouse infected with each strain was analyzed after 24 h. At this time, most foci lacked iNOS staining or contained only 1 to 3 iNOS^+^ cells (data not shown). We therefore focused further study on mice infected 48 h, when iNOS staining was prominent. IHC for iNOS^+^ cells showed distinct patterns from those described above for F4/80^+^ cells and provided insight into the structure of the inflammatory foci (Figure 5 and Table 2). iNOS was detected in cells that infiltrated the medium to large lesions; and in the interior of larger foci where staining for F4/80 was sparsely present, the iNOS-producing cells were prominent (Fig. 5A vs. 5C, Table 2 for iNOS, and Table S1 for F4/80). Conversely, ≥50% of small foci that tended to be covered with F4/80^+^ cells lacked iNOS^+^ cells (Table 2 for iNOS and Table S1 for F4/80). Further, the iNOS^+^ cells were distributed differently from PMNs (Fig. 5D and Table S1) and were mononuclear. Accordingly, iNOS-production could not be assigned to either PMNs or F4/80^+^ cells.

**Table 2:** Distribution of iNOS^+^ cells in inflammatory foci after infection with thermally preinduced *Y. pestis.*

iNOS^+^ Cell Distribution within Foci^a^														
		Foci	Small Foci^b^				Medium Foci^b^				Large Foci^b^			
Dose; Time; (No. Exps)	*Y. pestis*	Total No.	Cent.^c^	Inv.^c^	Edge^c^	None^c^	Cent.	Inv.	Edge	None	Cent.	Inv.	Edge	None
10^4^ 48 h (2^d^)	Parent	117	30% (6)	5% (1)	15% (3)	50% (10)	**21% (5)**	**33% (8)**	**21% (5)**	**25% (6)**	44% (32)	44% (32)	12% (9)	0% (0)
	Δ*yopM-1*	168	17% (1)	0% (0)	17% (1)	67% (4)	**54% (21)**	**25% (10)**	**13% (5)**	**8% (3)**	41% (51)	50% (62)	8% (10)	0% (0)

a Within each focus size, the percent having each distribution of stained cells is given, and the actual number of foci in each category is in parentheses.

b Foci were measured in their largest aspect and classified as small (<50 µm), medium (50 to 100 µm), and large (>100 µm).

c The presence of stained cells within the central part of the focus identified that lesion as having a Central (Cent.) distribution. This distribution tended to have iNOS^+^ cells scattered over the focus, often resembling a lattice. If the cells were seen only in a ring around the perimeter extending inward not more than 25% of the diameter of the focus, the distribution was designated as Edge. iNOS^+^ cells also demonstrated an “invasive” localization (Inv.), appearing internal to the outer 25% of diameter as well as in the edge but not in the center. Foci with no iNOS^+^ cells are designated “None”.

d Livers from 3 mice per strain were analyzed in one experiment; 2 mice per strain were analyzed in the other. The distributions of iNOS^+^ cells for medium foci between infections by the two *Y. pestis* strains were significantly different by Fisher's exact test (two-tailed p = 0.043; calculated using the online tool at quantitativeskills.com/sisa/statistics/fiveby2.htm). The distributions for small and large foci did not differ significantly.

The iNOS^+^ cells could often be seen to have numerous thin processes (illustrated in Fig. 5B) and likely represented iDCs, which are known to lose expression of the F4/80 surface marker previously present on their precursor MOs [26] and are known to appear in liver and spleen by d 2 of infection [19]. The distributions of these cells did not differ significantly between infections by the two *Y. pestis* strains within small and large foci. This accounts for the majority of foci analyzed (76% for Δ*yopM-1* and 87% for the parent strain). For the medium-sized foci, those due to the Δ*yopM-1* strain tended more to an invasive distribution of iNOS^+^ cells and less frequently lacked these cells than foci due to parent *Y. pestis*. This observation is tantalizing, because iDCs are important in conditioning the local inflammatory environment to promote activation of macrophages and PMNs and could contribute to the lower net growth of the mutant compared to the parent strain seen by d 2 of infection. However, the difference was only just below the cut-off for statistical significance and did not persist or intensify as foci became large. We suspect it is not biologically significant. The NO produced within inflammatory foci was not responsible for the growth difference between parent and Δ*yopM-1 Y. pestis*, because both parent and Δ*yopM Y. pestis* strains grew to ca. 10-fold higher numbers in livers of mice lacking iNOS (B6.129P2-*nos2^tm1Lau^*/J) (data not shown), showing that NO per se has an equally negative influence on the growth of both strains in liver.

Taken together, the histological observations at 48 h p.i. with ∼10^4^ thermally-induced *Y. pestis* found foci uniformly or centrally dominated by PMNs. KC/MOs had disappeared from the centers of at least half of medium-sized foci, and only weakly staining ones remained at the edges of large foci. Cells that could be iDCs had developed at the periphery and were penetrating the interior. Their outside-toward-in distribution suggests that their precursors derived from blood and not from endogenous liver F4/80^+^ cells that appeared early in infection in the interiors of foci. Dying cells were present in the centers or scattered uniformly throughout the foci at d 2 p.i., with significantly greater numbers of these in foci due to the parent strain. This finding represents a YopM-related phenotype: YopM delivered to inflammatory cells in the centers of foci resulted in cell death. This would be a direct effect of YopM on the cells that receive it, as we previously found that YopM's effect on its target cell does not involve a diffusible/mobile mediator in liver [19]. It is worth noting that hepatocytes undergo apoptosis during the process of coagulative necrosis due to inflammation in both infective and non-infective liver damage [27], [28]. Coagulative necrosis is distinctly more evident in the case of infection by parent *Y. pestis* than the Δ*yopM* strain by d 3 p.i. (Fig. 2 A and C) and correlated with the bacterial burden. On d 3, when viable numbers of the parent strain are significantly higher than than those of the mutant (Fig. 1, Day 3, closed symbols). However, at d 2 p.i., when the TUNEL studies were done, lesions due to the two strains did not show this large a difference in character, likely because the numbers of the two strains were more similar at that time (Fig. 1, Day 2, closed symbols; and data not shown for the experiment of Fig. S1). Accordingly, although we have not directly excluded a contribution from dying hepatic cells to the TUNEL staining, we believe this was not responsible for the differences we found. We hypothesize that the cells directly affected by YopM in liver are the KCs that are present in centers of foci as well as PMNs, and that this alters the inflammatory character of the focus in favor of survival and growth of the parent strain in the face of immigrating iDCs.

### Tests with caspase-3^−/−^ mice revealed a host factor with which YopM might act

**Figure 6:**
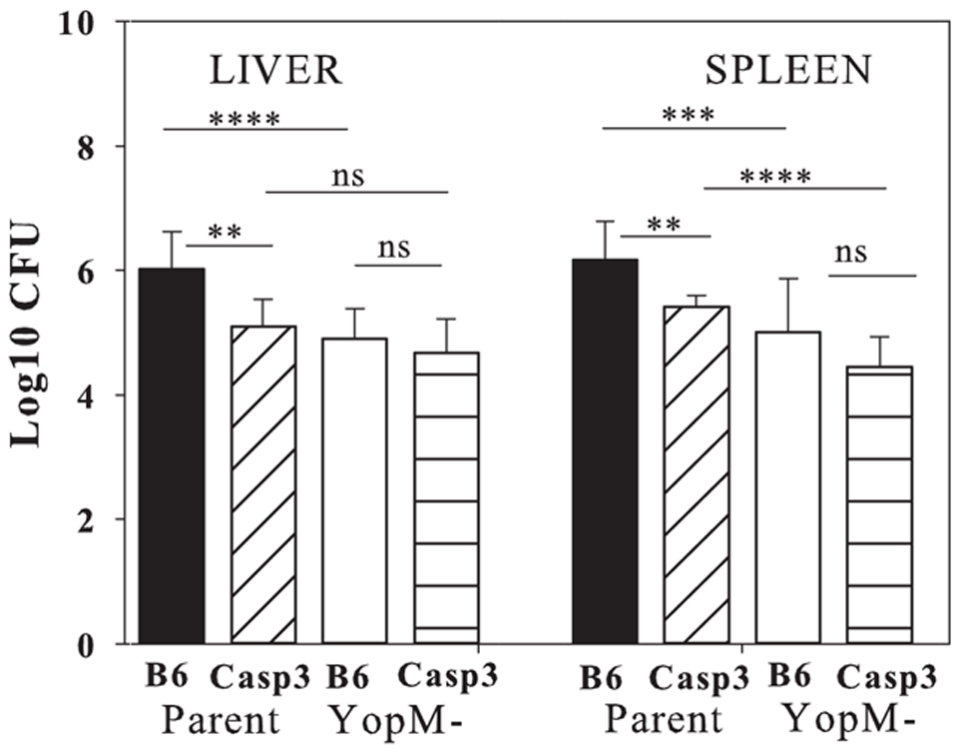
Tests with mutant strains of mice revealed a host factor with which YopM might act. Viable bacterial numbers for parent and Δ*yopM-1 Y. pestis* were assayed on d 3 in livers and spleens of mice from two experiments in which mice were infected with 500 parent or Δ*yopM-1 Y. pestis* grown at 28°C. Data for Casp3^−/−^ mice were pooled and represent responses in 8 (parent strain) or 12 (Δ*yopM-1* strain) mice per datum point. Data for B6 mice were pooled from 6 experiments and represent responses in 15 to 18 mice per datum point. The bars give geometric means and the error bars indicate the standard deviations. Significance of differences was assayed by the Student's two-tailed unpaired *t* test. *, P<0.05; **, P<0.01; ***, P<0.001; ****, P<0.0001.

We tested for a role of caspase-3, an “executioner” cysteine protease that mediates apoptosis, because of the correlation between the presence of YopM in the infecting strain and greater cell death in liver. We used a low IV dose (100 to 500 bacteria) of 28°C grown *Y. pestis* as in previously published experiments with mice ablated for different innate immune cells [17]–[19] and analyzed CFU in liver and spleen at d 3 p.i. when differences between parent and Δ*yopM-1 Y. pestis* numbers are large in wildtype mice. Figure 6 shows that in livers of these mice, growth of parent *Y. pestis* was restricted and resembled that of the *ΔyopM-1* strain, which was not altered by the absence of caspase-3. Growth of the parent strain was also inhibited in spleens of the Casp3^−/−^ mice, whereas the *ΔyopM-1* strain was not affected significantly; however, there remained some difference in viable numbers between parent and mutant strains in spleens of Casp3^−/−^ mice. This pattern of growth inhibition for the parent strain but not the Δ*yopM-1* mutant in knockout mice is distinct from that for loss of a target cell for YopM such as PMNs or a downstream molecular target such as caspase-1 [10], [19], where growth of the Δ*yopM-1* mutant is preferentially enhanced. These findings represent the discovery of an effector arm of YopM's pathogenic mechanism: YopM requires the presence of caspase-3 to function in both organs. Caspase-3 could be viewed as a cofactor for YopM. Loss of a cofactor molecule in effect inactivates YopM, and YopM+ bacteria would grow like the YopM- strain, while YopM- bacteria that lacked YopM in the first place would not be affected by absence of a host cofactor. Apparently, without caspase-3, an anti-bacterial host function is active against the parent as well as Δ*yopM-1* yersiniae. We hypothesize that in liver, this function would be activated PMNs. Although the bacteria also bind to KC/MOs which do disappear from centers of enlarging foci, the death of these cells could mainly be due to YopJ, which is known to cause apoptosis in MΦs [29]. In contrast, PMNs do not undergo apoptosis in response to YopJ [30]; hence YopM's apoptosis-inducing effect may be manifested most strongly on PMNs in liver as indicated previously by cell-depletion studies [19]. In spleen, YopM inhibits recruitment of iDCs, presumably in addition to promoting apoptosis of PMNs; hence YopM retains partial function there in the absence of caspase-3.

**Figure 7:**
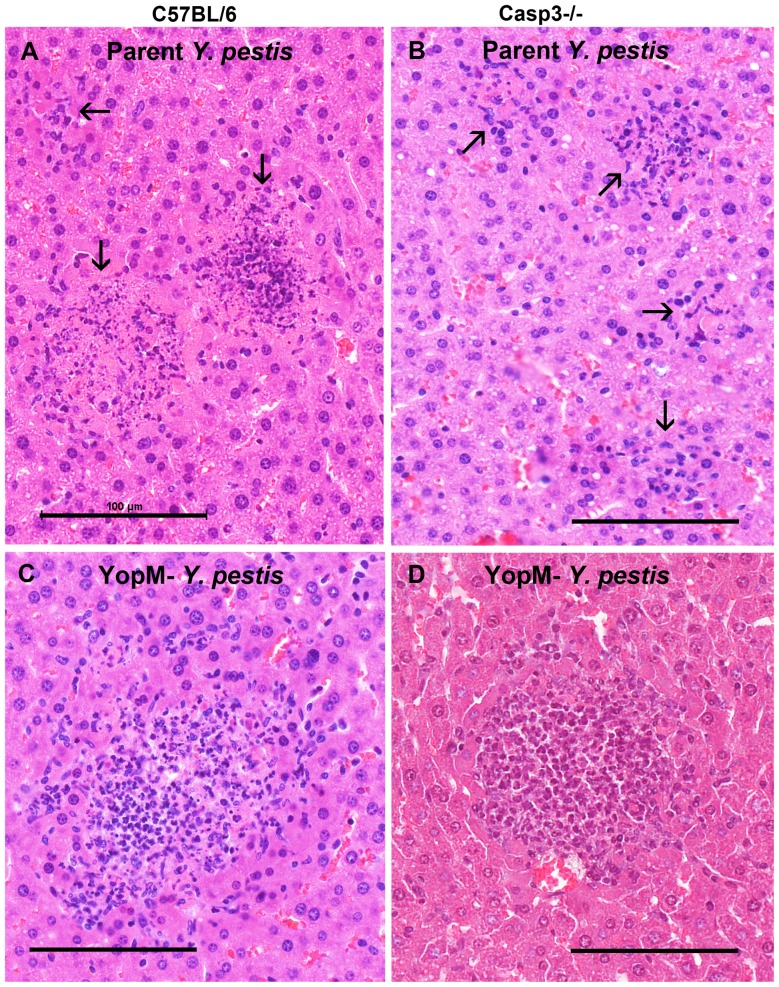
Typical lesions in livers from B6 and Casp3^−/−^ mice infected 3 d with a low dose of 28°C-grown parent and Δ*yopM-1 Y. pestis*. B6 and B6.129S1-*Casp3^tm1Flv^*/J knockout mice lacking active caspase-3 were infected IV with 100 to 500 parent or Δ*yopM-1 Y. pestis* grown at 28°C. On d 3 p.i., livers from mice infected by each *Y. pestis* strain in two independent experiments were recovered and sections were stained with H&E (11 *Casp3* ^−/−^ and 6 B6 mice infected by each bacterial strain in two experiments were examined). Typical lesions in mice that had the same CFU per liver as the means in Figure 6 are illustrated. Top panels (A, B), mice infected by parent *Y. pestis* (2×10^6^ CFU and 10^5^ CFU in the respective mice). Arrows point to foci. Bottom panels (C, D), mice infected with the Δ*yopM-1* strain (4×10^4^ CFU and 2×10^4^ CFU in the respective mice. The bars all represent 100 µm.

Figure 7 illustrates foci in mice that had bacterial burdens equal to the mean CFU values of Figure 6. Panels A and C show the difference in control B6 mice infected by parent and Δ*yopM-1 Y. pestis* where there is a 100-fold difference in bacterial burden in the presence of PMNs (documented also in [18]): the centers of foci in livers of control B6 mice infected with parent *Y. pestis* contained noticeably greater numbers of fragmented and condensed nuclei than foci due to the Δ*yopM-1* strain. In contrast, inflammatory cells in livers of Casp3^−/−^ mice infected by the two strains showed a less striking difference, with many intact nuclei remaining, even in the infection by parent *Y. pestis* (Fig. 7B and D). The decreased bacterial burden of the parent strain in Casp3^−/−^ mice indicates that the virulence of the parent *Y. pestis* was decreased in these mice. The B6 mice, with>10^6^ CFU per liver, would all have died had we waited several more days, whereas only 1 Casp3^−/−^ mouse of the 6 analyzed in full and 2 of 6 more from a pilot experiment had such high CFUs in these experiments. In such instances, the foci in the Casp3^−/−^ mice resembled those of Fig. 7A (B6 mice), with only scattered bits of inflammatory cells remaining (data not shown). This situation did not occur in infections by the Δ*yopM* mutant, and it is possible that the parent strain, despite its reduced virulence, still was more virulent than the Δ*yopM-1* strain in Casp 3^−/−^ mice. Accordingly, YopM may also have caspase-3-independent virulence effects, in agreement with the finding in Fig. 6 of greater viable bacterial numbers in spleens of Casp3^−/−^ mice infected by the parent strain than by the Δ*yopM-1* strain.

## Summary and Further Discussion

This study sought improved understanding of how YopM exerts its anti-host effect in liver, where PMNs are critical cells that YopM undermines, and YopM acts directly on cells without involvement of a diffusible or mobile mediator. KCs lining the sinusoids function to bind and clear LPS and bacteria from blood: they have multiple receptors for polysaccharides that bind bacteria to their surfaces [31], [32]. However, as exemplified by studies with *Listeria*, *Salmonella*, *Klebsiella*, and *Escherichia*, early bacterial killing in liver during systemic infection is largely accomplished by PMNs that are rapidly attracted from the circulation by Il-1β and IL-6 produced by KCs [31]. Our histological studies revealed that the bacteria are associated with myeloid cells from 17 h to 2 d p.i.; and by d 2 p.i. the presence of YopM in the infecting bacteria resulted in increased death of inflammatory cells over that seen in its absence. We speculate that this results from direct binding of the bacteria with delivery of Yops into PMNs and macrophages.

In both spleen and liver, caspase-3 was identified as a co-factor or effector in YopM's action: caspase-3 function was required for YopM to have its growth-promoting effect on the bacteria. This novel finding implicates the existence of an anti-bacterial target against which YopM directs caspase-3. Our data did not imply that YopM modulates caspase-3 activity: rather that caspase-3 is required for YopM to have its effect. Nonetheless, the presence of YopM in the infecting *Y. pestis* was reflected in greater numbers of dead inflammatory cells. This suggests that YopM acts directly or indirectly with or downstream of activated caspase-3. In macrophages, YopJ's inhibition of NF-κB and MAPKK kinase signaling indirectly provides the caspase-3 activation that YopM requires for its function [33], [34]. In PMNs, caspase-3-dependent apoptosis is a consequence of stimulation, but apoptosis is delayed by multiple factors within a proinflammatory milieu [35]. Further, YopJ does not cause apoptosis in PMNs, and several *Y. pestis* virulence properties inhibit major inducers of apoptosis: the Pla protease degrades Fas ligand, and YopH, YopE, and LcrV inhibit phagocytosis, the respiratory burst, and production of large amounts of TNFα [5], [30], [36]. It is tempting to speculate that YopM functions in a pathway that tips the balance toward caspase-3 activation and death of PMNs. Because PMNs otherwise limit growth of *Y. pestis* in liver, a function of YopM to promote apoptosis of these cells is a plausible pathogenic mechanism.

The pathway(s) through which YopM functions are beginning to be assembled for macrophages. YopM invokes novel signaling pathways by recruiting and activating kinases such as RSK1 and PRK2 that do not normally work together [7], but the downstream effects of these interactions have not yet been determined. In the context of stimulatory LPS on the bacteria, YopM also prevents maturation of the NLRP3, NLRC4, and NLRP1 inflammasomes by preventing the activation of caspase-1 in macrophages [10]. One player in this mechanism is the scaffold protein IQGAP1, which YopM was recently shown to bind [37]. YopM's function in PMNs has not previously been examined. It is intriguing that in PMNs IQGAP1 functions in a complex with the chemokine receptor CXCR2 to mediate effects of CXCL1/KC (CXCL8/IL-8 in human PMNs) and CXCL2/MIP-2α [38]. These effects include delaying both spontaneous and TNFα-induced apoptosis [39], [40]. Future studies are needed to learn whether YopM binds IQGAP1 and inhibits CXCR2 signaling in PMNs.

## Supporting Information

Figure S1 **Similar amounts of TUNEL staining were present in spleens of mice infected with parent and Δ** ***yopM-1 Y. pestis*** **.** B6 mice were infected IV with 10^5^ parent or Δ*yopM-1 Y. pestis* grown at 28°C and recovered at d 2 p.i. Sections of spleens and livers were stained by the TUNEL reaction for cells undergoing DNA fragmentation (bright green fluorescence). Panels A and B illustrate the similar amounts of TUNEL^+^ nuclei due to the two *Y. pestis* strains in spleens. Panels C and D illustrate the greater amount of TUNEL staining in liver foci due to the parent strain (panel C) than to the Δ*yopM-1* mutant (panel D), as was seen in the experiments described in the main manuscript that used 10^4^ thermally pre-induced bacteria. The DAPI-stained nuclei in the same liver sections are shown below their TUNEL counterparts in panels E and F. The foci in both liver and spleen were quantified for percent of focus area that contained green fluorescence brighter than background, and the results are mentioned in the text. In all panels the bars represent 100 µm.
(TIF)

Table S1 **Distribution of Ly6G^+^ and F4/80^+^ Cells in foci after infection with thermally preinduced** ***Y. pestis*** **.**
(DOCX)
