# Supplementary figures and images for: Caspase-3 Mediates the Pathogenic Effect of Yersinia pestis YopM in Liver of C57BL/6 Mice and Contributes to YopM's Function in Spleen
Source: PLoS One. 2014 Nov 5;9(11):e110956. doi: 10.1371/journal.pone.0110956 (PMC4220956; doi:10.1371/journal.pone.0110956)

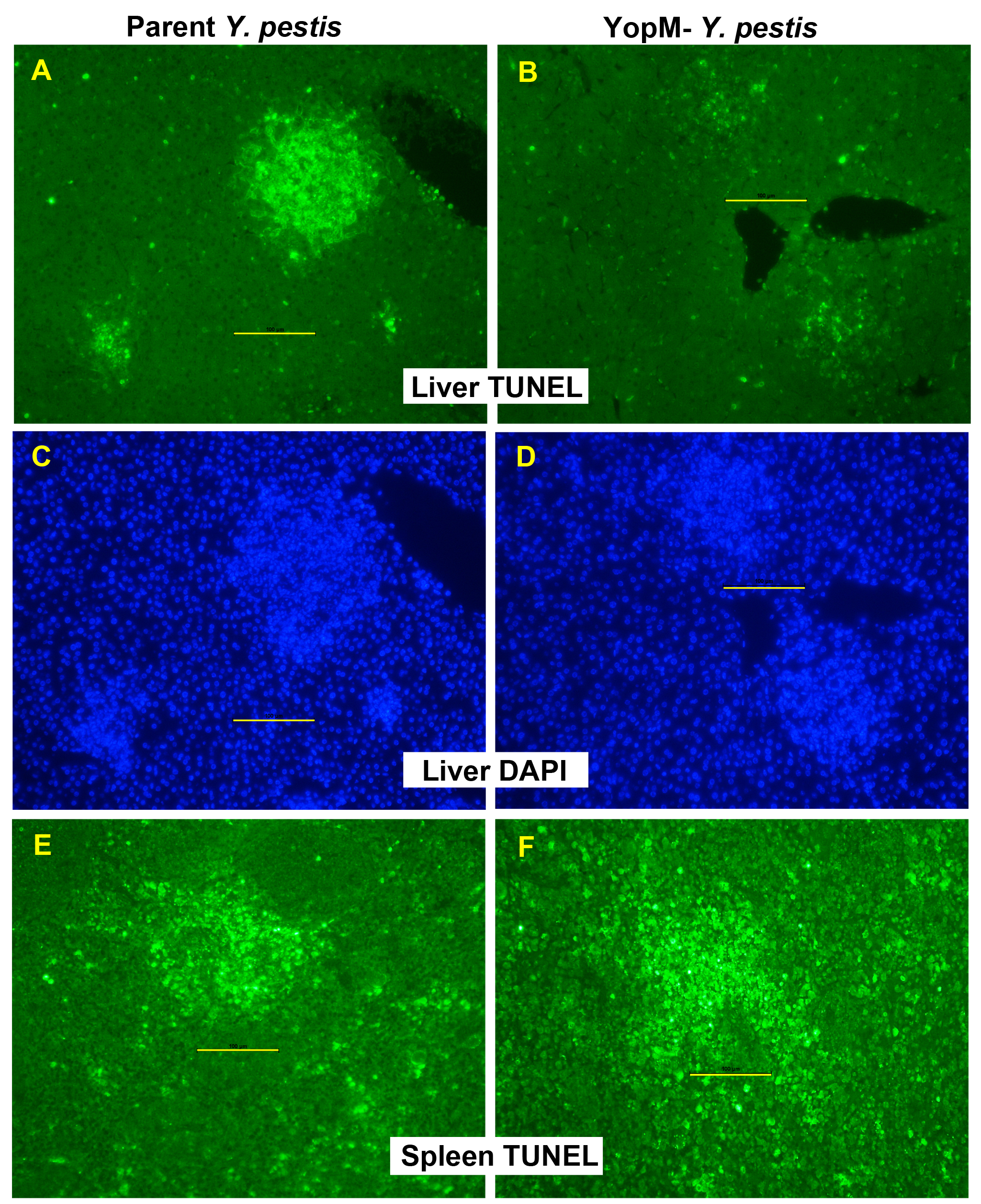

Supplement: Figure S1 — Similar amounts of TUNEL staining were present in spleens of mice infected with parent and Δ yopM-1 Y. pestis . B6 mice were infected IV with 105 parent or ΔyopM-1 Y. pestis grown at 28°C and recovered at d 2 p.i. Sections of spleens and livers were stained by the TUNEL reaction for cells undergoing DNA fragmentation (bright green fluorescence). Panels A and B illustrate the similar amounts of TUNEL+ nuclei due to the two Y. pestis strains in spleens. Panels C and D illustrate the greater amount of TUNEL staining in liver foci due to the parent strain (panel C) than to the ΔyopM-1 mutant (panel D), as was seen in the experiments described in the main manuscript that used 104 thermally pre-induced bacteria. The DAPI-stained nuclei in the same liver sections are shown below their TUNEL counterparts in panels E and F. The foci in both liver and spleen were quantified for percent of focus area that contained green fluorescence brighter than background, and the results are mentioned in the text. In all panels the bars represent 100 µm. (TIF) [file pone.0110956.s001.tif]
