# Supplementary material for: Caspase-3 Mediates the Pathogenic Effect of Yersinia pestis YopM in Liver of C57BL/6 Mice and Contributes to YopM's Function in Spleen
Source: PLoS One. 2014 Nov 5;9(11):e110956. doi: 10.1371/journal.pone.0110956 (PMC4220956; doi:10.1371/journal.pone.0110956)
Supplement: Table S1 — Distribution of Ly6G+ and F4/80+ Cells in foci after infection with thermally preinduced Y. pestis . (DOCX) [file pone.0110956.s002.docx]

Table S1. Distribution of Ly6G^+^ and F4/80^+^ Cells in foci after infection with thermally preinduced *Y. pestis*

|  |  | | **Foci** | **Small Foci^a^** | | **Medium Foci^a^** | | **Large Foci^a^** | |
| --- | --- | --- | --- | --- | --- | --- | --- | --- | --- |
| **Dose; infection duration; (No. Exps)** | ***Y. pestis*** | | **Total No.** | **Central^b^** | **Edge^b^** | **Central** | **Edge** | **Central** | **Edge** |
| **Ly6G^+^ Cell Distribution within Foci^c^** | | | | | | | | | |
| 10^7^ 17 h (1) | Parent | 32 | | 90% (18) | 10% (2) | 92% (11) | 8% (1) | 0% (0) | 0% (0) |
|  | Δ*yopM-1* | 24 | | 100% (16) | 0% (0) | 100% (8) | 0% (0) | 0% (0) | 0% (0) |
| 3 x 10^4^ 48 h (1) | Parent | 207 | | 98% (96) | 2% (2) | 93% (79) | 7% (6) | 75% (18) | 25% (6) |
|  | Δ*yopM-1* | 94 | | 100% (19) | 0% (0) | 94% (31) | 6% (2) | 98% (41) | 2% (1) |
| **F4/80^+^ Cell Distribution within Foci^c^** | | | | | | | | | |
| 10^7^ 17 h (1) | Parent | 42 | | 73% (16) | 27% (6) | 6% (1) | 94% (17) | 0% (0) | 100% (2) |
|  | Δ*yopM-1* | 40 | | 71% (15) | 29% (6) | 11% (2) | 89% (16) | 0% (0) | 100% (1) |
| 10^4^ 24 h (1) | Parent | 46 | | 94% (16) | 4% (1) | 74% (17) | 26% (6) | 50% (3) | 50% (3) |
| 10^4^ 48 h (1) | Parent | 42 | | 70% (14) | 30% (6) | 33% (6) | 67% (12) | 0% (0) | 100% (4) |
|  | Δ*yopM-1* | 18 | | 100% (7) | 0% (0) | 50% (2) | 50% (2) | 0% (0) | 100% (7) |

^a^ Inflammatory foci were measured for their largest aspect and classified as small (< 50 μm), medium (50 to 100 μm), and large (> 100 μm).

^b^ The presence of stained cells within the central part of the focus identified that lesion as having a Central distribution. If the cells were seen only in a ring around the perimeter extending inward not more than 25% of the diameter of the focus, the distribution was designated as Edge. Ly6G^+^ cells in small foci were uniformly distributed over the foci. A notable percentage of medium and large foci had Ly6G^+^ cells restricted to a central core surrounded by an annulus of Ly6G^-^ cells (14% and 72% of medium and large foci, respectively, for the parent *Y. pestis* and 35% and 59%, respectively, for the Δ*yopM-1* strain). Foci with a central distribution of stained cells tended to have F4/80^+^ cells all over the focus (not just at the center).

^c^ Within the indicated focus sizes, the percent having each distribution of stained cells is given, and the actual number of foci in each category is in parentheses.
